# Transcriptomic Profiling Reveals Altered Expression of Genes Involved in Metabolic and Immune Processes in NDV-Infected Chicken Embryos

**DOI:** 10.3390/metabo14120669

**Published:** 2024-12-02

**Authors:** Malarmathi Muthusamy, Kannaki T. Ramasamy, Sunday Olusola Peters, Srinivasan Palani, Vasudevan Gowthaman, Murali Nagarajan, Sivakumar Karuppusamy, Vasanthakumar Thangavelu, Thiruvenkadan Aranganoor Kannan

**Affiliations:** 1 Department of Animal Genetics and Breeding, Veterinary College and Research Institute, Tamil Nadu Veterinary and Animal Sciences University (TANUVAS), Namakkal 637002, India; 2 Indian Council of Agricultural Research—Directorate of Poultry Research, Hyderabad 500030, India; tr.kannaki@icar.gov.in; 3 Department of Animal Science, Berry College, Mount Berry, GA 30149, USA; speters@berry.edu; 4 Department of Veterinary Pathology, Veterinary College and Research Institute, Tamil Nadu Veterinary and Animal Sciences University (TANUVAS), Namakkal 637002, India; srinivasan.p@tanuvas.ac.in; 5 Poultry Disease Diagnosis and Surveillance Laboratory, Tamil Nadu Veterinary and Animal Sciences University (TANUVAS), Namakkal 637002, India; gowthaman.v@tanuvas.ac.in; 6 Alambadi Cattle Breed Research Centre, Tamil Nadu Veterinary and Animal Sciences University (TANUVAS), Dharmapuri 635111, India; murali.n@tanuvas.ac.in; 7 Faculty of Food and Agriculture, The University of the West Indies, St. Augustine 999183, Trinidad and Tobago; sivakumar.karuppusamy@sta.uwi.edu; 8 Veterinary University Training and Research Centre, Karur 639006, India; vasanthakumar.t@tanuvas.ac.in

**Keywords:** NDV, chicken, immune genes, metabolic genes, APOC3, ALDOB, BPI, TRIM3 HSP

## Abstract

Objective: The poultry industry is significantly impacted by viral infections, particularly Newcastle Disease Virus (NDV), which leads to substantial economic losses. It is essential to comprehend how the sequence of development affects biological pathways and how early exposure to infections might affect immune responses. Methods: This study employed transcriptome analysis to investigate host–pathogen interactions by analyzing gene expression changes in NDV-infected chicken embryos’ lungs. Result: RNA-Seq reads were aligned with the chicken reference genome (Galgal7), revealing 594 differentially expressed genes: 264 upregulated and 330 downregulated. The most overexpressed genes, with logFC between 8.15 and 8.75, included C8A, FGG, PIT54, FETUB, APOC3, and FGA. Notably, downregulated genes included BPIFB3 (−4.46 logFC) and TRIM39.1 (−4.26 logFC). The analysis also identified 29 novel transcripts and 20 lncRNAs that were upregulated. Gene Ontology and KEGG pathways’ analyses revealed significant alterations in gene expression related to immune function, metabolism, cell cycle, nucleic acid processes, and mitochondrial activity due to NDV infection. Key metabolic genes, such as ALDOB (3.27 logFC), PRPS2 (2.66 logFC), and XDH (2.15 logFC), exhibited altered expression patterns, while DCK2 (−1.99 logFC) and TK1 (−2.11 logFC) were also affected. Several immune-related genes showed significant upregulation in infected lung samples, including ALB (6.15 logFC), TLR4 (1.86 logFC), TLR2 (2.79 logFC), and interleukin receptors, such as IL1R2 (3.15 logFC) and IL22RA2 (1.37 logFC). Conversely, genes such as CXCR4 (−1.49 logFC), CXCL14 (−2.57 logFC), GATA3 (−1.51 logFC), and IL17REL (−2.93 logFC) were downregulated. The higher expression of HSP genes underscores their vital role in immune responses. Conclusion: Comprehension of these genes’ interactions is essential for regulating viral replication and immune responses during infections, potentially aiding in the identification of candidate genes for poultry breed improvement amidst NDV challenges.

## 1. Introduction

Newcastle Disease Virus (NDV) is critically linked to global food security, particularly in developing countries, where poultry serves as a vital source of nutrition and income [1]. India’s total egg production for the fiscal year 2022–2023 was projected to reach 138.38 billion eggs. This included 118.16 billion eggs from commercial poultry and 20.20 billion eggs from backyard poultry. Additionally, poultry meat production was estimated at 4.995 million metric tons, which constituted 51.13% of the country’s total meat output (Source: BAHS Statistics 2023 DAHD, Government of India) [2]. India ranks third globally in egg production and fifth in chicken meat production (Source: FAO) [3] and has experienced significant changes in poultry disease patterns due to a considerable increase in the poultry population and shifts in husbandry practices. These changes have led to both direct and indirect economic losses [4,5]. The rapidly growing poultry industry faces numerous challenges, particularly in managing disease problems. Worldwide, the poultry sector incurs substantial economic losses annually due to diseases [5]. Among these, Newcastle Disease is particularly impactful, causing significant production losses and high mortality rates [6,7]. This disease poses a major threat to the poultry industry, leading to considerable economic setbacks for poultry farmers [4,5]. A study revealed that Newcastle Disease (ND) outbreaks in India resulted in economic losses of INR 3,719,223 (approximately USD 45,000) across 58 farms [8]. Furthermore, Iranian government estimates suggested that ND could cause annual economic losses exceeding INR 80 million (around USD 1 million) across affected farms due to increased mortality and reduced productivity. Overall, the economic impact of ND on Iran’s poultry industry is estimated to be approximately USD 288.49 million annually [9]. Further, the implementation of Newcastle Disease Virus (NDV) vaccination among rural smallholder chicken farmers in developing and low-income countries is significantly influenced by socioeconomic factors [10]. Limited financial resources restrict farmers’ ability to afford vaccinations, while inadequate access to veterinary services and vaccines further complicates the situation. Additionally, traditional farming practices and poor infrastructure hinder the adoption of modern vaccination and biosafety measures, with community norms and regulatory environments shaping farmers’ willingness to engage in these interventions [10,11].

However, Newcastle Disease (ND) is a highly infectious viral infection that affects poultry, resulting in substantial financial losses for the poultry industry globally. ND has the ability to quickly spread throughout avian communities, destroying whole flocks of chickens [12]. Although indigenous breeds have greater tolerance to various diseases compared to commercial chicken breeds, native backyard chicken breeds, such as Aseel and Kadaknath, are still affected [13,14,15]. Aseel and Kadaknath are prominent Indian chicken breeds recognized for their resilience in challenging agroclimatic conditions [16]. The Aseel breed, a game bird from South India, is celebrated for its robust muscle development. In contrast, the Kadaknath is a black chicken native to North India. Both breeds are well known for their disease tolerance and significantly contribute to the genetic diversity of the chicken population [17]. Research has shown that both breeds possess heat and disease tolerances, making them valuable in local poultry farming [18]. The Aseel is particularly noted for its high-quality meat and has a reputation in cockfighting, while the Kadaknath is primarily raised for its unique black flesh. Their adaptability and genetic traits enhance their value in diverse farming environments across India [16]. Modern high-efficiency growth techniques in commercial poultry farming have put selection pressure on immunological competence. This has led to intensive breeding practices that produce chicken lines more susceptible to common infections [19]. Research indicates that while fast-growing commercial breeds excel in growth and production rates, they frequently exhibit lower immunocompetence compared to indigenous chicken breeds. For example, studies have shown that broilers selected for high growth rates often experience higher mortality rates due to infectious diseases, as their immune responses may not keep pace with their rapid growth [20,21]. Now, the best ways to manage ND infection are vaccination and biosafety measures. A comprehensive understanding of the molecular mechanisms by which the host immune system responds to ND infection is essential for developing new prevention and treatment strategies for ND [18]. So, a new approach, such as a transcriptomic study in chickens, particularly in connection with NDV infection, would provide considerable insights into host immunological responses and genetic heterogeneity. For instance, research found 1386 differentially expressed genes (DEGs) in chicken thymus tissues 96 h after infection, indicating immune-related genes that were both upregulated and downregulated [22]. Understanding the biological mechanisms triggered by infection requires this kind of research. Another study on transcriptomic responses in the tracheal epithelial layer to NDV infection under heat stress revealed breed-specific differences in immune response timing and strength between indigenous and Leghorn chickens. Indigenous chickens showed an earlier pro-inflammatory response [12]. Further analyses, including Gene Ontology (GO) and Kyoto Encyclopedia of Genes and Genomes (KEGG), identified enriched immune pathways, providing insights into host defense mechanisms and pathogenesis [22,23]. Additionally, the embryonic stage of immune system development in chickens is crucial for understanding their responses to NDV infection. The regulation of developmental genes during embryogenesis plays a pivotal role in shaping the functionality of the immune system. By day 10 of incubation, the chicken embryo begins to develop its immune system, and by day 18, it is considered immunocompetent, capable of mounting both innate and adaptive immune responses to pathogens, including NDV [24,25,26]. It is essential to comprehend how the sequence of development affects biological pathways and how early exposure to infections might affect immune responses. Hence, the current study demonstrates the use of the chicken embryo to understand the sequential regulation of genes that govern basic cellular programming and signaling pathways, going beyond mere morphological observations.

## 2. Materials and Methods

### 2.1. Ethics Statement

This study was conducted in strict accordance with the guidelines established by the Committee for Control and Supervision of Experiments on Animals (CCSEA), a statutory body under the Department of Animal Husbandry and Dairying, Ministry of Fisheries, and the Department of Biotechnology, Ministry of Science and Technology, Government of India. The experiments involving virulent Newcastle Disease Virus (NDV) were performed in Class 2-B2 cabinet facilities. All procedures were conducted with prior approval and supervised by the Institutional Biosafety Committee (IBSC) at VCRI, Namakkal (Approval Lr. No. 1764/VCRI-NKL/IBSC/2022, dated 11 May 2022, by the Dean, VCRI, Namakkal), and the Institutional Animal Ethical Committee (IAEC; Project Proposal No. 09/VCRI-NKL/2023, dated 8 September 2023) of TANUVAS, Veterinary College and Research Institute, Namakkal, Tamil Nadu, India.

### 2.2. Experimental Birds and Sample Collection

Here, 18 SPF chicken embryos (19 days old), comprising 6 Aseel, 6 Kadaknath, and 6 Commercial Layer White Leghorn, were obtained from the Department of Poultry Science at VCRI in Namakkal for a viral challenge study. In addition to a control group of 3 Aseel, 3 Kadaknath, and 3 White Leghorn, the remaining 3 Aseel, 3 Kadaknath, and 3 White Leghorn embryos were inoculated with a lentogenic-B1 strain of live Newcastle Disease Virus (NDV) at a dosage of 50 percent embryo infective dose (10^6^ EID50). Following inoculation, the embryos were incubated in an egg incubator at a temperature of 38 °C, with relative humidity maintained between of 65 and 75 percent, until tissue harvesting at 24 h post-infection.

### 2.3. RNA Isolation and Quality Check

Lung tissue was collected from NDV-infected and control chicken embryos at 24 h post-infection. Tissues were immediately stored in RNAlater^®^ (ThermoFisher, Vilnius, Lithuania) at −20 °C until RNA isolation. Total RNA was extracted using the Trizole method, using the RNAiso Plus, M/s Takara, (DSS Takara Bio India Pvt Ltd., Bangalore, India). The amount of extracted RNA was measured using a nano-dropper, and RNA samples were combined at an equimolar ratio to form one pool for each breed of treated samples (three samples from each breed combined), as well as a pool for each breed of control samples. In order to verify the quality, a 2% electrophoresis gel was used in the laboratory to view the integrity of the extracted RNA. Samples were then shipped to be sequenced after being sealed on dry ice. Clevergene Biocorp Pvt. Ltd., Bangalore, India, performed the RNA sequencing. RNA was measured using the Qubit^TM^ RNA HS Assay Kit, in accordance with the manufacturer’s instructions, on the Qubit 3.0 Fluorometer (Thermofisher Scientific, Vilnius, Lithuania). Using RNA screen tape, the 4150 TapeStation system (Agilent, Bangalore, India) was used to evaluate the integrity of RNA. RNA with a RIN greater than 7 was deemed intact and proceeded to further processing for the development of an RNA library. The estimated library fragment size comprised a wide peak with an average size of 350 bp and a range of 200–700 bp. The sample passed the quality check with more than 3 ng/µL, and more than 6 nM for the molarity concentration.

### 2.4. Sequencing and Data Analysis

The sequence data were generated using the Illumina NovaSeq 6000 sequencer and with the Adapter Sequence, P7 adapter read1—AGATCGGAAGAGCACACGTCTGAACTCCAGTCA, and P5 adapter read2—AGATCGGAAGAGCGTCGTGTAGGGAAAGAGTGT (Illumina, San Diego, CA, USA). The generated RNA sequence data were analyzed, and the analysis workflow, focused on differential expression and Gene Ontology/pathway enrichment, began with data quality control (QC) to ensure the integrity of raw sequencing data, using tools such as MultiQC. Next, high-quality reads are aligned to a reference genome or transcriptome during the read mapping step, using STAR for alignment. Following this, expression estimation was performed to quantify transcript abundance from the aligned reads, with gene expression levels calculated in R. Differential expression analysis was then conducted using DESeq2 to identify transcripts that showed significant differences in expression between infected and control embryos. Finally, Gene Ontology (GO) and pathway enrichment analyses were performed to interpret the biological functions, processes, and pathways that were overrepresented in the differentially expressed gene set. Sequence reads were processed to remove adapter sequences and low-quality bases using fastp 0.20 (default version) [27]. Clean data for downstream analysis required quality assurance and FASTQ file preparation [28].

The QC passed reads were mapped onto the indexed bGalGal1.mat.broiler.GRCg7b_genome using the STAR (2.7.10b_alpha) aligner [29]. The distribution of genes, exons, and transcripts per chromosome and genes was examined using the Chi-square test, and *p* < 0.05 was deemed significant. Gene level expression values were obtained as read counts using feature counts [30]. Principal component analysis (PCA) is a powerful statistical technique used to analyze and visualize the patterns of gene expression data. For differential expression analysis, the biological replicates were grouped as control and infected groups. Differential expression analysis was carried out using the DESeq2 (version 1.4.0) [31] package after normalizing the data using the relative log expression normalization method. Genes with absolute log_2_ fold change ≥1 and adjusted *p*-value ≤ 0.05 were considered significant.

### 2.5. Functional Analysis

Further, the set of transcripts identified from the sequence data were analyzed for functional significance, and GO, KEGG, and gene cluster comparisons were performed using bioinformatic tools with the R package (version 4.2.1), such as Cluster Profiler, GOplot, and Pathview. GOplot, Pathview, and ShinyGO are complementary tools for functional analysis. GOplot visualizes gene expression with GO and pathway enrichment results, while Pathview maps differential expression onto biological pathways [32,33,34]. In short, within the examined gene set, we looked at whether functional terms or pathways were statistically significant and linked to at least two genes. The Benjamini–Hochberg method employs a significance threshold of *p*-values < 0.05 to modify the calculated *p*-values. Gene Ontology and enrichment analyses were performed by using the online tool ShinyGO 0.80 (http://bioinformatics.sdstate.edu/go/, accessed on 17 August 2024) [35]. ShinyGO offers quick, interactive enrichment analysis with minimal setup and enhances the interpretation of functional enrichment data. The online free SRplot platform was used for graphing and data visualization [36].

### 2.6. Protein–Protein Interaction (PPI)

Protein–protein interactions (PPIs) were obtained using the STRING (Search Tool for the Retrieval of Interacting Genes/Proteins) version 12.0 online bioinformatics tool (https://version-12-0.string-db.org/cgi/, accessed on 16 July 2024) [37]. In order to ascertain the functional relationship between the genes related to metabolism, cell cycle, immunity, and mitochondria, DEGs (with corrected *p*-values of ≤0.05) annotated for the KEGG pathway were utilized to establish a network of close interactions among this gene set based on databases of predicted and experimental protein interactions [37]. We eliminated PPIs based on high confidence ratings of less than 0.7, and disconnected nodes were found. In order to identify a certain number of clusters based on their centroids, k-mean clustering was also performed.

### 2.7. Quantitative Real-Time PCR (RT-qPCR) Validation

To validate the RNA-Seq results by RT-qPCR, five differentially expressed genes (TLR2, TRIM14, IL1R2, NOS1, and GATA3) were randomly selected, and primers were designed using Primer-Blast. Three biological replicates were conducted for each breed. Total RNA was extracted from the lungs of chickens using RNAiso Plus, M/s Takara (DSS Takara Bio India Pvt Ltd., Bangalore, India), according to the manufacturer’s protocols, and the concentration and purity of RNAs were measured using NanoDrop. The cDNA was synthesized from total RNA using an iScript cDNA Synthesis Kit (Bio-Rad, Hercules, CA, USA), according to the manufacturer’s protocol, which follows a method of reverse transcription (RT) with a random primer. The details of all the designed primers are provided in Table S1 (Supplementary File S1). The relative expression of specific gene mRNA was quantified as per the method in [38] using a real-time thermal cycler (Roche LightCycler^®^ 96) with Bio-Rad Universal SYBR Green Master Mix (Bio-Rad Laboratories India Pvt. Ltd, Chennai, India). RT-qPCR was performed with the following thermocycling conditions: an initial cycle at 95 °C for 10 min, 40 cycles at 95 °C for 15 s, 60 °C for 20 s, and 72 °C for 20 s, followed by a 72 °C elongation for 60 s. β-Actin was used as a housekeeping gene to normalize the expressions of mRNA. The alteration in mRNA levels of each gene in NDV-infected embryos was quantified as a fold change using the 2^−ΔΔCt^ method [39], in comparison to the non-infected control.

## 3. Results

### 3.1. RNA-Seq Data Analysis

The average number of reads obtained from lung RNA sequencing was 25 million, and the Q20 and Q90 rates showed average matching call accuracy of 95% and 90%, respectively. On average, 20 million of the 25 million reads made it past quality control. An analysis of the sequence revealed that its average GC content was 54%. A summary of data quantity and quality is presented in Tables S2 and S3 (Supplementary File S1). On average, 99.84% of the reads aligned with the reference genome (GRCg7b). According to read alignment statistics, on average, 88%, 2%, and 10% of the sequences were identified as unique mapped, multi-mapped, and unmapped sequences, respectively. A total of 30,108 genes were selected from the reference, of which 17,410–20,332 genes were expressed in all samples (Table S4).

### 3.2. Principal Component Analysis (PCA)

A principal component analysis (PCA) plot of normalized gene expression data was used to perform data reduction when dealing with a high number of variables (Figure S1). In this analysis, PC1 captured 100% of the variance, while PC2 captured 0% of the variation. This result is quite straightforward but unusual, indicating a very specific data structure. This pattern was observed when comparing control and infected samples within a breed across all three breeds individually. Whereas, while considering all breeds together and comparing the control and infected groups, PC1 (x-axis, 35.74% variance) captured the largest variance in the data, and PC2 (y-axis, 30.06% variance) captured the second largest variance in the data, slightly less than PC1. The control samples (1C, 2C, and 3C) were clustered together on the right side of the plot, represented by the red color. The treated samples (1T, 2T, and 3T) were clustered together on the left side of the plot. There was a clear separation between the control and treated samples along both PC1 and PC2. Together, PC1 and PC2 accounted for 65.80% of the total variance (30.06% + 35.74%). This indicates that the principal components effectively captured the significant differences between the control and treated groups.

### 3.3. Identification and Characterization of Transcripts and Differential Expression Analysis

**Figure 1:**
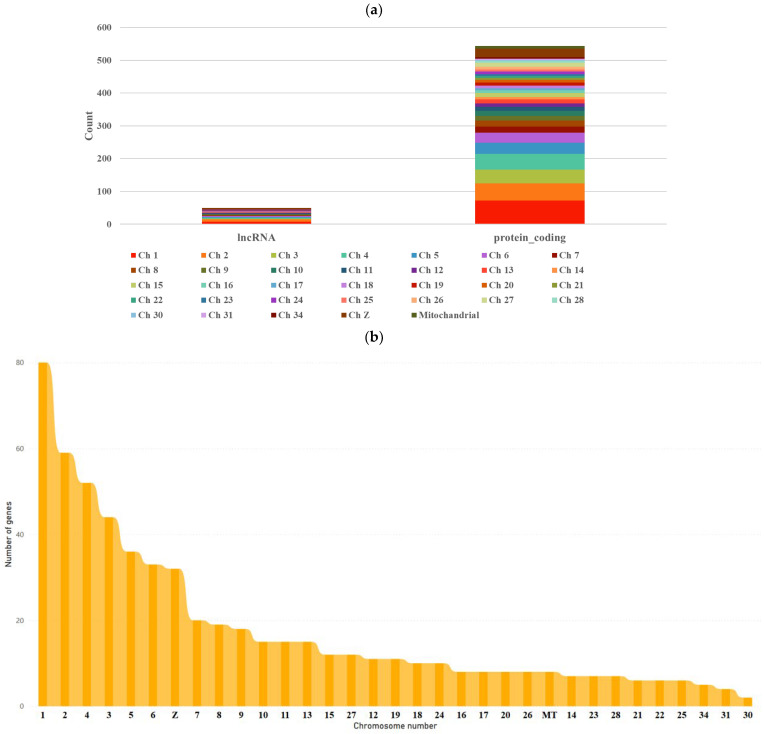

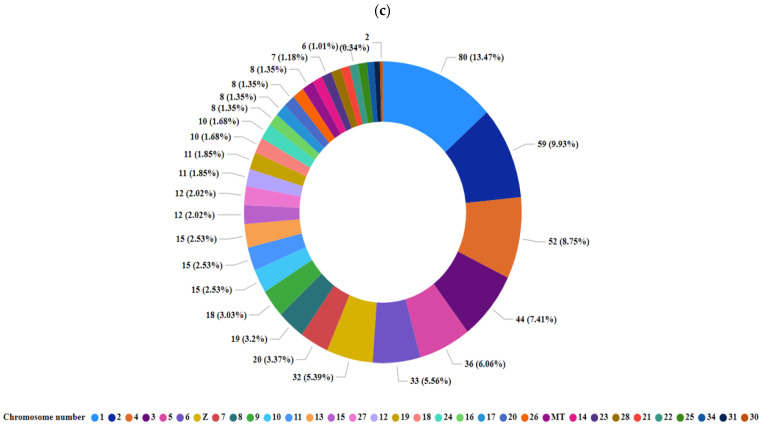
The proportion of differently expressed genes from the respective chicken chromosomes. (**a**) The amount of lnRNA and protein-coding RNA transcripts, (**b**) the overall total amount of RNA from each chromosome, and (**c**) the quantity of transcripts expressed from each chromosome.

In the differential expression analysis, biological replicates were categorized into control and treated groups. Among the 23,400 genes tested for significance by comparing control and treated samples within the same breed, significant differences were found. However, when all control samples were compared to all treated samples across breeds, 594 genes exhibited significant differential expression (*p* < 0.01). Of them, 544 protein-coding RNA transcripts were expressed from different chromosomes, and 50 lnRNA transcripts were detected (Figure 1, Table S5 (Supplementary File S1)). Chromosome number one expressed the most transcripts, making up 13.47% of all transcripts, with 72 protein-coding RNA and 8 lnRNA transcripts. After the first chromosome, the highest percentage of gene expression was noticed from the second (9.93%), fourth (8.75%), third (7.41%), fifth (6.06%), sixth (5.56%), and Z (5.39%) chromosomes. Genes expressed from the remaining chromosomes accounted for less than 4% (Figure 1).

**Figure 2:**
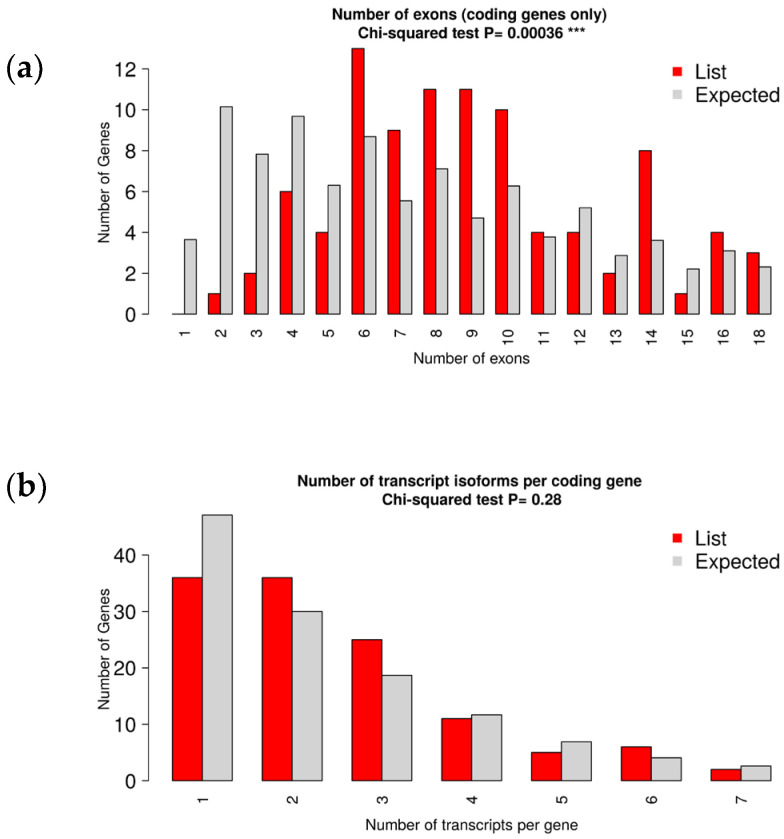
Bar diagram illustrating the proportionate number of exons (**a**) and transcripts (**b**) per gene.

**Figure 3:**
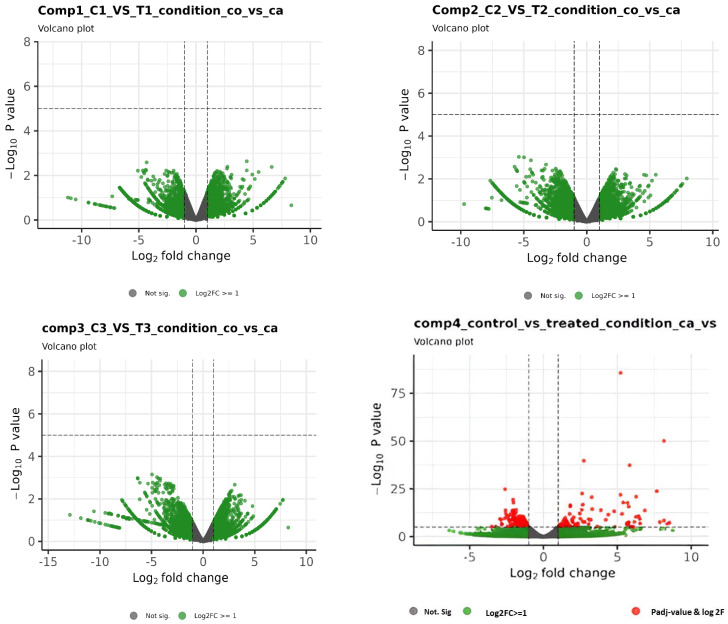
Volcano plot showing differential expression profiles of genes. Red indicates absolute log2 fold change ≥1 and adjusted *p*-value ≤ 0.01. As the first three comparisons did not have any significantly expressed genes, the graphical results are provided only for the fourth comparison (1C, 2C, and 3C vs. 1T, 2T, and 3T).

According to the Chi-square test, there was no significant difference in the distribution of query genes across the chromosomes (*p* = 0.37). The number of exons per gene showed extremely significant (*p* < 0.01) variations (Figure 2). Findings revealed that the majority of the genes contained six exons and transcribed one to two RNA transcripts. The quantity of transcripts for each gene was also not observed to vary significantly (*p* > 0.05; Figure 3).

**Figure 4:**
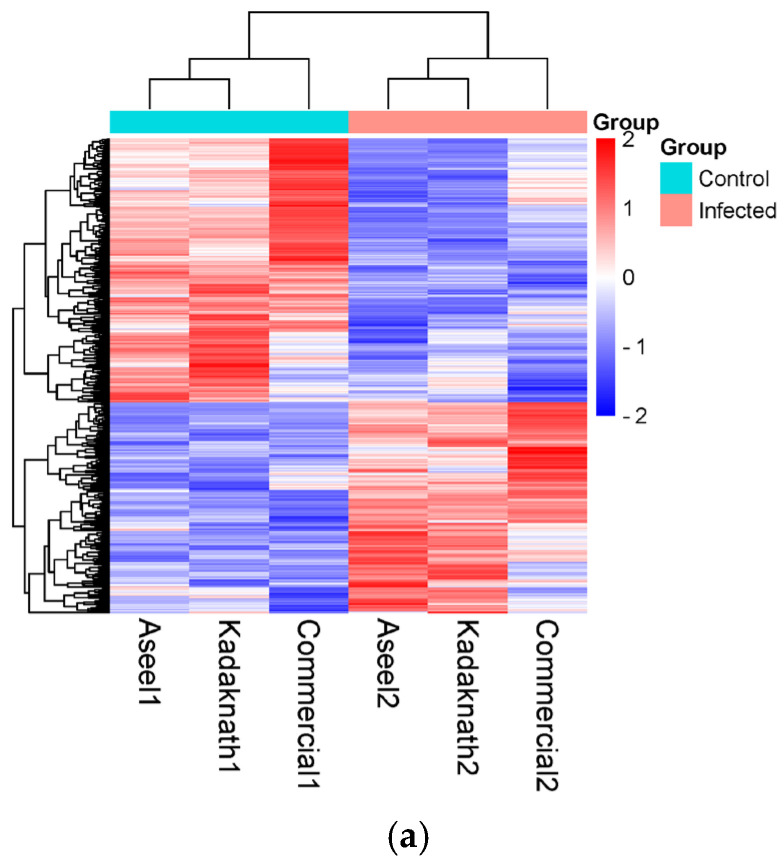

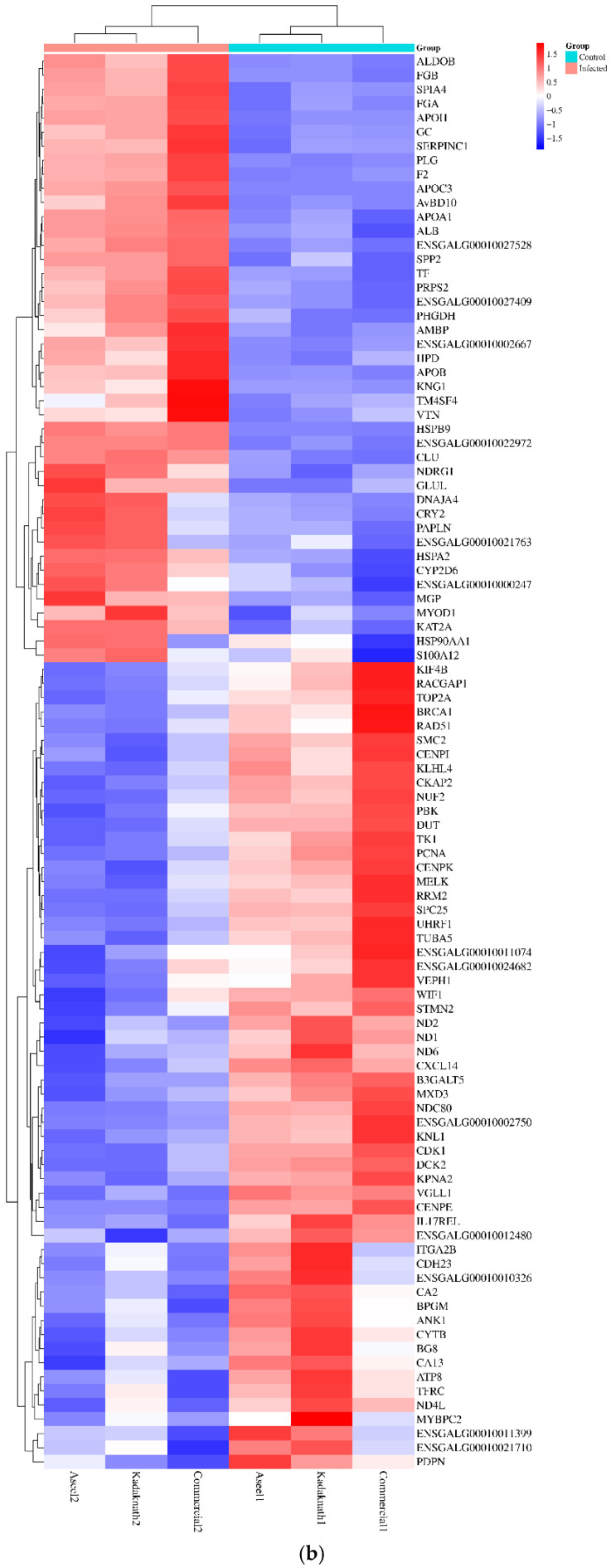
Hierarchical cluster and expression profile of (**a**) 594 differently expressed genes (*p* < 0.002) and (**b**) the highly significant (*p* < 10 ^−7^) differentially expressed top 100 genes across the samples. The heat maps were generated from the normalized expression values of each sample for a given gene.

Out of 594 differentially expressed genes, 264 genes (44%) were upregulated, while 330 genes (56%) were downregulated (Supplementary File S2). Similar results were found in a volcano plot for gene expression analysis, and the gene expression level was two times higher in the treated group compared to the control group (Figure 3). A heat map was generated for the expression profile of the significant differentially expressed top 100 genes across the samples (Figure 4) to show the breed-specific pattern of gene expression. A few genes’ expression was shown as exceptionally up- or down-regulated compared to the majority, which could be of particular interest for further investigation, as they may play key roles in the biological processes under study. The highly and significantly (*p* < 0.05) overexpressed genes (log2 fold change) in infected chicken embryonic conditions included ALB, C8A, FGG, PIT54, FETUB, APOC3, FGA, SPIA1, AvBD13, APOH, AMBP, TTR, PLA2G12B TLR4, TLR2, TLR21, IL1R2, IL22RA2, HSP90AA, HSPB9, and HSPB8. All these genes were found to be expressed with a log2 fold change greater than 8. Additionally, 29 novel transcripts and 20 lncRNAs were found to be significantly upregulated in infected chicken embryos.

Among these, the top 12 highly expressed transcripts were ENSGALG00010016177, ENSGALG00010017501, ENSGALG00010006269, ENSGALG00010027528, ENSGALG00010002667, ENSGALG00010022972, ENSGALG00010014764, ENSGALG00010022012, ENSGALG00010017606, ENSGALG00010021130, ENSGALG00010022479, and ENSGALG00010000247. Furthermore, clusters of genes with comparable expression patterns were visible on the heat map, which may point to co-regulation or shared functional pathways. In all three breeds, the majority of the genes exhibited uniform changes in expression in response to infection.

Infected samples had significantly (*p* < 0.01) downregulated levels of 331 genes in total. Results showed that the quantities of mRNA for BPIFB3 (−4.46 logFC), TRIM39.1 (−4.26 logFC), MAP3K7CL (−3.4 logFC), IL17REL (−2.93 logFC), KHDRBS2 (−2.68 logFC), KCNH5 (−2.64 logFC), VGLL1 (−2.63 logFC), CA2 (−2.6 logFC), CXCL14 (−2.57 logFC), and SLC25A48 (−2.54 logFC) genes were significantly low in infected lungs. In infected chicken embryos, 34 new genes were under-expressed, and 10 novel transcripts were identified to be strongly downregulated in infected samples (ENSGALG00010029558, ENSGALG00010011074, ENSGALG00010000768, ENSGALG00010028733, ENSGALG00010028337, ENSGALG00010011399, ENSGALG00010018325, ENSGALG00010011368, ENSGALG00010017833, and ENSGALG00010027164).

Infected embryos also showed 28 under-expressed and 20 overexpressed lnRNAs. Out of 28 lnRNAs, 10 were found highly under-expressed (ENSGALG00010030044, ENSGALG00010012473, ENSGALG00010007497, ENSGALG00010010093, ENSGALG00010028388, ENSGALG00010002237, ENSGALG00010011328, ENSGALG00010019663, ENSGALG00010014138, and ENSGALG00010011830) in infected embryos.

Genes associated with various metabolic activities, such as APOC3, PLA2G12B, FTCD, ENSGALG00010027528, and ADH6 transcripts, exhibited significantly higher expression levels in infected embryos, with log fold changes (logFC) of 8.16, 6.58, 5.85, 5.83, and 5.65, respectively, compared to healthy embryos. Conversely, several transcripts, including BPGM (−2.06 logFC), CA13 (−2.10 logFC), TK1 (−2.11 logFC), ATP6V0D2 (−2.44 logFC), LCT (−2.52 logFC), and CA2 (−2.60 logFC), were significantly under-expressed.

Additionally, genes involved in cell cycle regulation and DNA replication, such as NDC80 (−2.08 logFC), E2F2 (−2.20 logFC), CCNE2 (−2.35 logFC), MCM (−1.81 logFC), CDC45 (−1.68 logFC), and CDK1 (−1.85 logFC), were also found to be significantly under-expressed in infected embryos. Likewise, mitochondrial genes, including ND1 (−1.29 logFC), ND2 (−1.35 logFC), ND4 (−1.10 logFC), ND5 (−1.21 logFC), and ND6 (−1.44 logFC), were found to be downregulated. In contrast, immune-related genes, such as IL1R2 (3.15 logFC), TRIM14 (1.24 logFC), TLR2 (2.79 logFC), TLR21 (1.22 logFC), and TLR4 (1.22 logFC), showed increased expression levels in infected embryos.

### 3.4. RNA-Seq Data Validation by RT-qPCR

**Figure 5:**
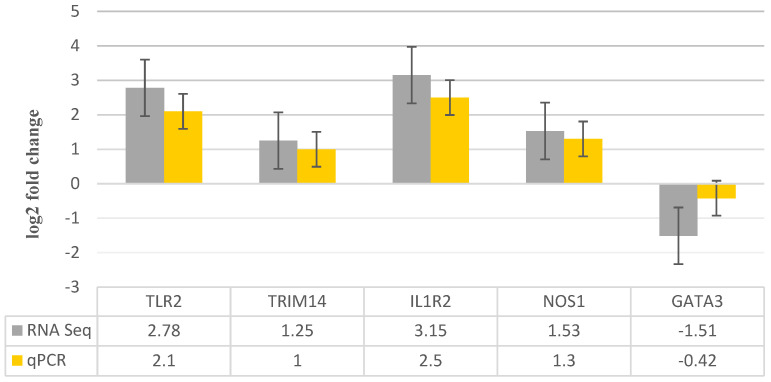
The bar graph depicts the five transcripts that were chosen at random and validated by RT-qPCR. The fold change indicates the variation in transcript amounts between infected and control samples.

Five mRNAs that were differently expressed were subjected to RT-qPCR in order to further verify the correctness of the sequencing findings. When comparing RT-qPCR data to RNA-Seq data, a comparable expression pattern was seen, as shown in Figure 5. However, there were differences seen in various techniques, which are due to the inherent characteristics of these methodologies.

### 3.5. Functional Analysis

**Figure 6:**
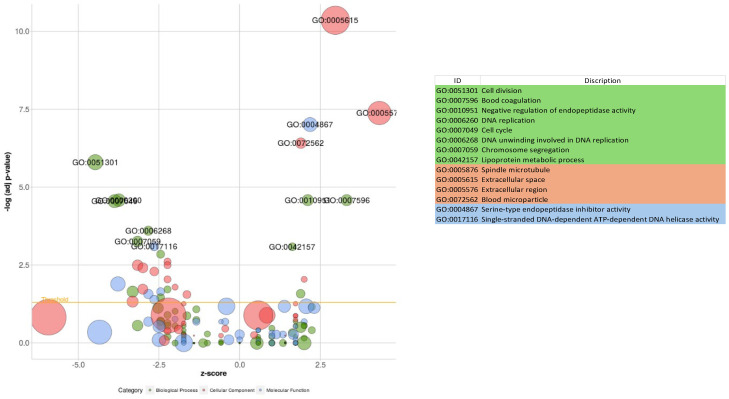
Control vs. treated groups (COMP4) bubble plot of the overrepresented GO terms using significantly expressed genes. The orange line represents the *p*-value threshold (Benjamini–Hochberg *p* < 0.05). The size of the bubble is proportionate to the number of genes involved in the GO term.

**Figure 7:**
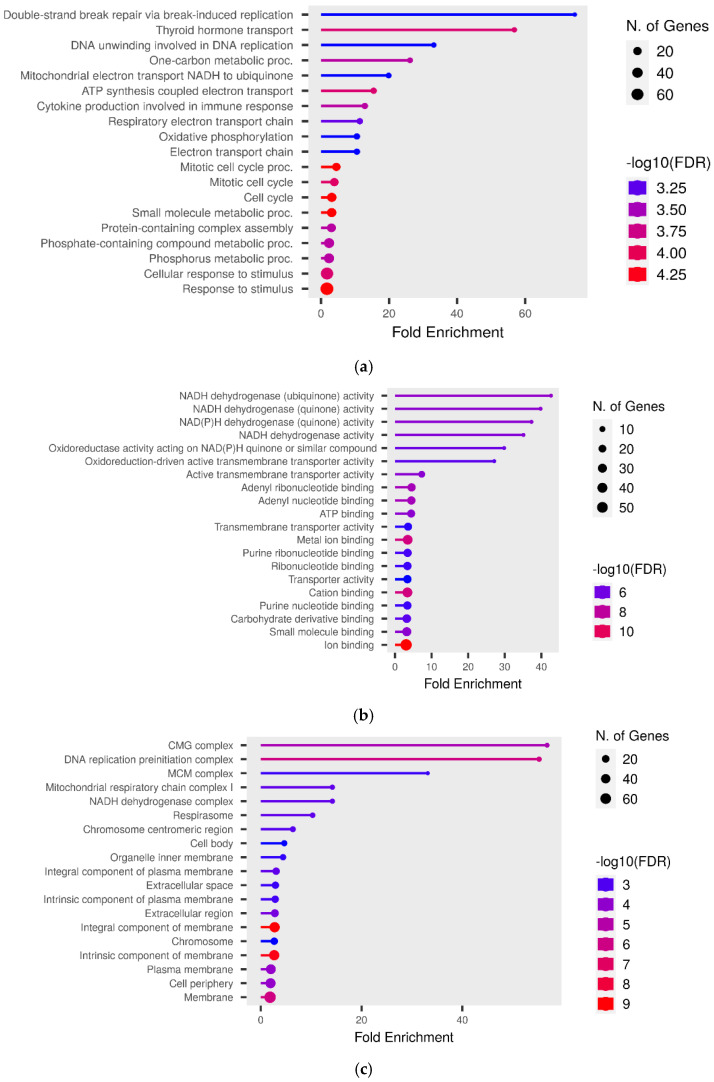
The Lollypop graph displays the results of the functional fold enrichment study conducted on the target genes of predicted known RNAs pertaining to various different (**a**) biological, (**b**) metabolic, and (**c**) cellular processes.

**Figure 8:**
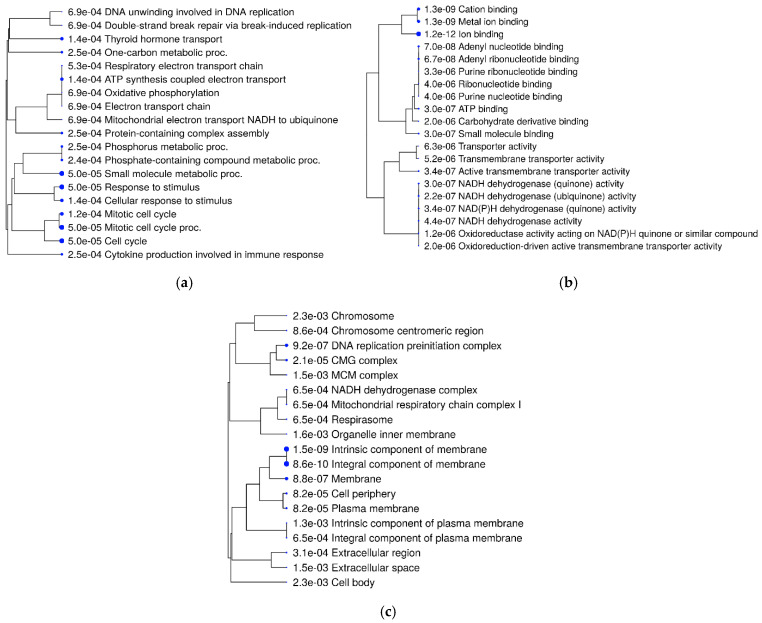
A hierarchical clustering tree of (**a**) biological, (**b**) metabolic, and (**c**) cellular processes, summarizing the correlation among significant pathways listed in the enrichment tab. Pathways with many shared genes are clustered together. Bigger dots indicate more significant *p*-values.

The GO over-representation analysis revealed several significant GO terms across different categories (*p* < 0.05; Figure 6). The most enriched GO terms included “extracellular space” (GO:0005615), with 71 genes and 2.61-fold enrichment within the cellular component category, “serine-type endopeptidase inhibitor activity” (GO:0004867), with 17 genes and 7.97-fold enrichment in the molecular function category, and “cell division” (GO:0051301), with 20 genes and 5.77-fold enrichment among other biological activities. The high fold enrichment and low adjusted *p*-values indicated robust associations that were unlikely to be due to chance. Notably, the “blood microparticle” term exhibited a high fold enrichment, indicating a strong association with the gene list. Further analysis showed that the “cytokine production involved in immune response” had a −log10 (FDR) of 3.25, which provided a clearer picture of the statistical significance with involvement of 20 genes. Additionally, it was also found that mitochondrial electron transport, specifically NADH to ubiquinone (GO:0006120), and mitochondrial respiratory chain complex I activity (GO:0032981) were affected, with 5 genes involved and fold enrichments of 6.69 and 4.6, respectively. This indicated an alteration in mitochondrial activity due to the infection. Enriched pathways and hierarchical clustering tree analysis revealed more significantly enriched gene sets, with greater gene overlap as well as a link between important pathways. Pathways that shared a large number of genes were grouped together (Figure 7 and Figure 8).

### 3.6. KEGG Pathway Analysis

**Figure 9:**
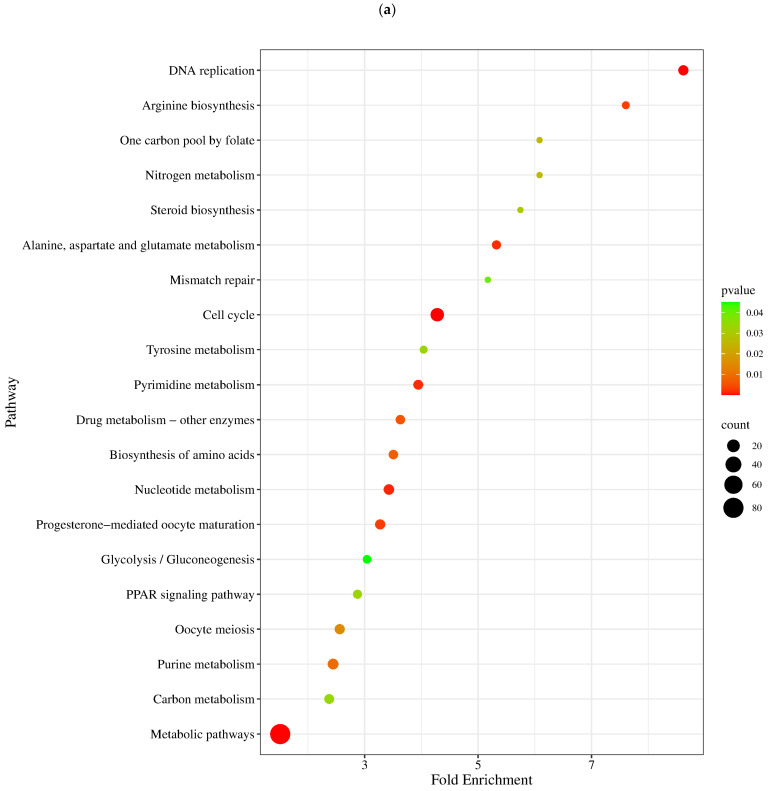

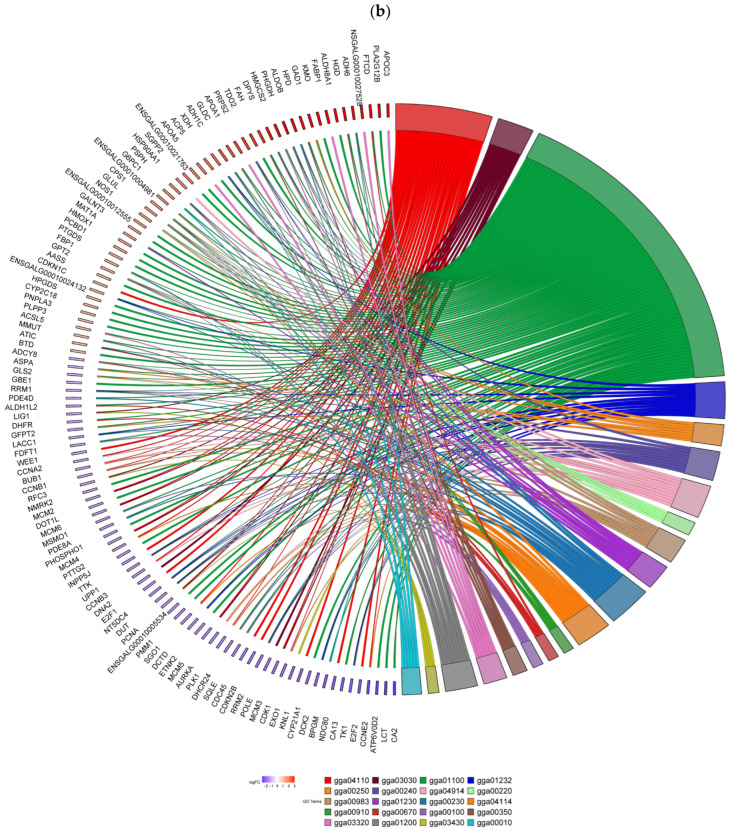
KEGG pathway enrichment analysis of transcripts with differential expression against NDV. (**a**) A bubble graphic illustrates the degree of enrichment and the number of genes in KEGG pathways. The top 20 significant KEGG keywords in metabolic processes are displayed in a chord plot (**b**).

Out of a total of 209 genes, 117 were found to be significantly involved (*p* < 0.05) in 20 KEGG pathways, with fold enrichment ranges from 1.5 to 8.6. Among these, the cell cycle (gga04110), DNA replication (gga03030), and metabolic (gga01100) pathways showed a particularly high level of significance (*p* < 0.001) and included a greater number of genes (80), all with a low false discovery rate (FDR < 0.001). Additionally, highly significant (*p* < 0.01) metabolic pathways were identified, including gga01232 (nucleotide metabolism), gga00250 (alanine, aspartate, and glutamate metabolism), gga00240 (pyrimidine metabolism), gga04914 (progesterone-mediated oocyte maturation), gga00220 (arginine biosynthesis), gga00983 (drug metabolism—other enzymes), gga01230 (biosynthesis of amino acids), and gga00230 (purine metabolism). Figure 9 shows the relationship bewteen KEGG terms, genes, and fold enrichment levels.

The analysis of metabolic pathway genes revealed significant alterations in gene expression in infected chicken embryos due to NDV infection. Several genes were markedly downregulated, including CA2 (−2.60 logFC), LCT (−2.52 logFC), ATP6V0D2 (−2.44 logFC), TK1 (−2.11 logFC), CA13 (−2.10 logFC), BPGM (−2.06 logFC), DCK2 (−1.99 logFC), CYP21A1 (−1.96 logFC), RRM2 (−1.75 logFC), SQLE (−1.62 logFC), and DHCR24 (−1.57 logFC). Conversely, a set of genes exhibited notable upregulation, including ACP5 (2.05 logFC), XDH (2.15 logFC), ADH1C (2.48 logFC), GLDC (2.52 logFC), PRPS2 (2.66 logFC), TDO2 (2.74 logFC), FAH (2.79 logFC), DPYS (2.96 logFC), HMGCS2 (3.04 logFC), PHGDH (3.07 logFC), ALDOB (3.27 logFC), HPD (3.30 logFC), GAD1 (3.82 logFC), KMO (3.84 logFC), ALDH8A1 (4.27 logFC), HGD (4.47 logFC), ADH6 (5.65 logFC), FTCD (5.85 logFC), and PLA2G12B (6.58 logFC). These findings highlighted significant disruptions in metabolic processes associated with NDV infection, underscoring the profound impact of the virus on embryonic metabolism. In the cell cycle pathway, a total of 24 genes were involved, with only CDKN1C showing upregulation (−1.21 logFC). All other genes were downregulated (−2.35 to −1.14 logFC), indicating that NDV infection led to disruptions in cell cycle regulation and growth in the infected embryos.

### 3.7. Protein–Protein Interaction (PPI)

**Figure 10:**
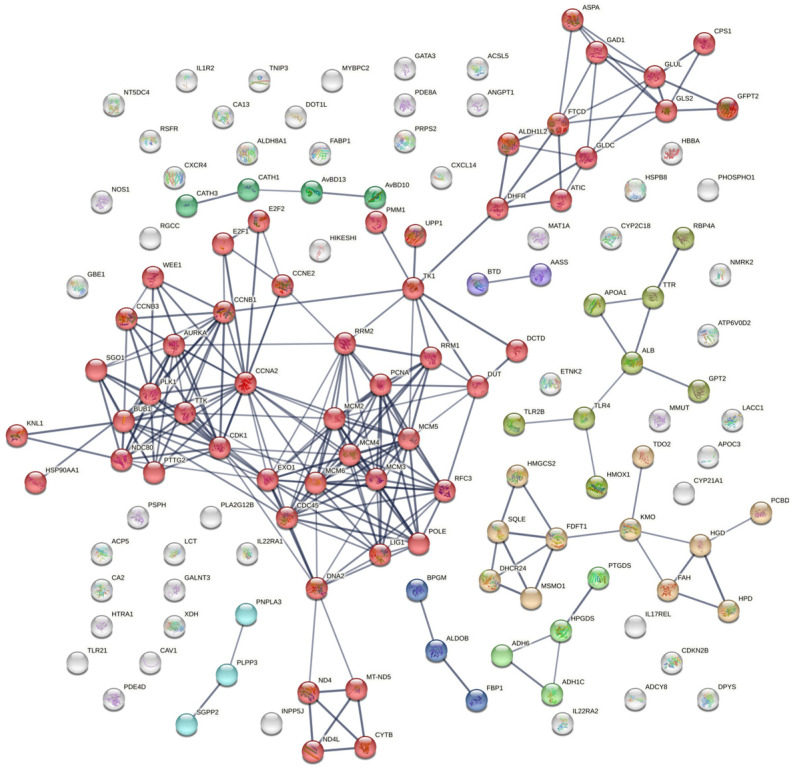
Potential networks of protein interactions encoded by genes related to different biological and cellular mechanics of cell functions. We drew the interaction networks using STRING functional protein association networks (https://string-db.org, accessed on 16 July 2024). Proteins with known or projected three-dimensional structures have clusters indicated by their color.

Protein–protein interactions (PPIs) were analyzed selectively for 150 genes using the STRING (version 12.0) online bioinformatics tool, with a minimum required interaction score set at the confidence level of 0.700. This analysis revealed that the proteins had more interactions among themselves than expected for a random set of proteins of the same size and degree distribution drawn from the genome. This enrichment indicated that the proteins were at least partially biologically connected as a group. The network consisted of 136 nodes and 244 edges, with an average node degree of 3.59. The average local clustering coefficient was predicted to be 0.452, and the expected number of edges in the PPI enrichment network was 46, with a *p*-value of less than 10^−16^ and FDR < 0.05. The k-mean clustering results showed that there were eight distinct clusters. A central area with a high density of connections may signify a fundamental cellular mechanism, such as transcription regulation or control of the cell cycle. Less connected peripheral nodes may be associated with more specialized roles or newly identified proteins, with few known interactions, as depicted in Figure 10 Edges represent protein–protein associations, i.e., proteins jointly contributed to a shared function—this does not necessarily mean they were physically bound to each other. The included genes, belonging to the RRM, CCN, TNF, HDP, CXC, IL, HSP, MCM, and mitochondrial genes, etc., were found to be significantly interacting with each other (*p* < 0.05). The module was significantly enriched for 198 biological processes, 3 molecular functions, and 9 cellular component GO terms, including regulation of metabolic, cell cycle, nucleic acid metabolism, innate, humoral, TLR signaling, and cytokine signaling pathways.

## 4. Discussion

The present study utilized a global transcriptome analysis to investigate molecular events and gene expression patterns in lungs of chicken embryos infected with Newcastle Disease Virus (NDV) across three different breeds. This approach allowed us to identify changes in gene expression linked to NDV infection and to examine candidate genes involved in the innate immune response of chicken embryos to the virus. By taking this comprehensive transcriptome-wide perspective, the study provided new insights into the host response to NDV infection and the complex interactions between the virus and its host. These findings contribute valuable information that can inform and guide future research efforts in this area. Our study investigated the dynamic gene expression changes induced in the lung by NDV at 24 hpi to understand the host response during viral infection in Aseel, Kadaknath, and commercial chicken embryos.

With the development of next-generation sequencing, transcriptome analyses have been performed for many bacterial and viral infections, such as *Mycoplasma gallisepticum*, *Pasteurella multocida*, laryngotracheitis, Duck Hepatitis A Virus, and Fowl Adenovirus [40,41,42,43,44,45].

The immune system of chickens has a direct impact on their health and plays a significant role in the farm economy, influenced by the genetic makeup of the flock [46]. RNA-Seq technology enables the detection of key genes associated with important traits, such as disease and heat resistance [47,48]. Consequently, high-throughput sequencing was conducted to assess the gene expression profiles of three chicken breeds with extreme phenotypes. This analysis aims to understand the potential differences in their immune and metabolic pathways that may be crucial in responding to Newcastle Disease viral infection [1,25].

### 4.1. Differential Gene Expression Analysis

RNA-sequencing studies showed that Chromosome 1 expressed the highest number of transcripts among the macrochromosomes in chicken embryos, underscoring its critical role in regulating gene expression during embryogenesis. Macrochromosomes, such as chr1, chr2, chr3, chr4, chr5, chr6, chr7, chr8, chr9, and chrZ, contain over 1000 genes [49,50]. Another study reported a strong correlation between the size of chicken chromosomes and the abundance of lncRNAs and mRNAs (r = 0.9850 and 0.9677, respectively) [49], suggesting that transcript distribution is proportional to chromosome size. This relationship contributes to higher transcriptional activity in larger chromosomes during the developmental stages of chicken embryos [51,52]. When comparing the infected embryos of White Leghorn, Aseel, and Kadaknath to their respective control uninfected counterparts, significant differences were observed in DEGs across the infected embryos at 24 h post-infection (hpi) within each breed. Similarly, when analyzing all infected embryos collectively against all uninfected embryos, regardless of breed, significant differences in DEGs were also identified. This suggests that a broader comparison reveals notable changes in gene expression associated with the infection.

At two days post-infection (dpi), a large number of DEGs were found in the lungs of NDV-infected Fayoumi chickens, with 122 DEGs being noticeably elevated. The C8A gene was significantly upregulated in infected embryos and encoded a protein that is part of the complement membrane attack complex (MAC). This complex plays a crucial role in the immune defense mechanism against invading microorganisms and infected host cells [53]. In a study similar to the one mentioned, researchers found that the genes FGG, FGA, TNIP3, and IL1R2 were predominantly involved in the immune response of broiler thymus tissue against lipopolysaccharide (LPS) challenge [54]. The cytokine-signaling-related gene, IL17REL, and the phagosome maturation pathway-related genes, NOX4, PRDX1, and RAB7B, are important immune-related genes [12]. Similar to our study, Rabiei et al. [55] reported that NDV-infected Ross broiler chickens exhibited differentially expressed genes (DEGs) in the spleen, which included 55 upregulated genes and 45 downregulated genes. In contrast, a previous study found 389 DEGs in the lungs of chicken embryos infected with *Mycoplasma gallisepticum*, where 96.14% of the genes were upregulated and 3.86% were downregulated [45]. The variations in differentially expressed genes (DEGs) between NDV and *Mycoplasma gallisepticum* infections can be attributed to several factors, including the nature of the pathogens, the mechanisms of host immune responses, variability in experimental design, and specific virulence factors [56]. Especially, NDV induces apoptosis, leading to a wider range of gene expression alterations, which includes both upregulation and downregulation of genes associated with stress responses, apoptosis, and immune regulation. This apoptotic response is significant, as it reflects the complex interactions between the virus and host cellular mechanisms during infection [55]. In another study, 564 differentially expressed lncRNAs were observed in chickens infected with the parasite *Eimeria tenella*. Of these, lncRNA BTN3A2 was revealed to control the inflammatory response to coccidia infection [57].

In recent years, long non-coding RNAs (lncRNAs) have garnered significant attention, with numerous studies revealing their crucial roles in various physiological and pathological processes across different species [58,59]. While the functions of many lncRNAs are well documented in humans and mice, with 17,948 and 13,186 lncRNA genes, respectively, identified in each species, research on lncRNAs in domestic animals, particularly chickens, is still in its early stages [60,61]. Only 4641 lncRNA genes were found in the Ensembl reference database in chickens, compared to 18,346 protein-coding genes [61]. It was also stated that, similar to other species, 79% of chicken lncRNAs are found in intergenic regions [62].

Numerous novel transcripts with unique expression patterns have been found in the infected chicken embryo. The ENSGALG00010002557 and ENSGALG00010002584 transcripts encode a protein with an Ig-like domain that binds to signal receptors and T cell receptors, playing roles in biological processes and the regulation of cytokine production, according to the PANTHER (Protein Analysis Through Evolutionary Relationships) database [63]. According to reports from the Ensembl database, 50 transcripts encoding long non-coding RNAs (lncRNAs) were identified, exhibiting differential expression in chicken embryos infected with Newcastle Disease Virus (NDV). This observation implies that these lncRNAs may actively participate in the immune system’s defense against NDV. Recent studies have underscored the critical role of lncRNAs in modulating antiviral immune responses. Upon viral infection, host cells initiate a signaling cascade through pattern recognition receptors (PRRs), which activates transcription factors and leads to the subsequent expression of cytokines and antiviral proteins. The differential expression of lncRNAs can significantly influence various aspects of the immune response, including the regulation of PRR-related signaling pathways and the production of interferons (IFNs) and other cytokines, which are essential for effective antiviral defense [64,65].

Similarly, numerous genes were shown to have changed expression in infected embryos, even after taking individual gene expression patterns into consideration. Particularly high levels of downregulation were found in the TRIM and BPIFB3 genes. The bactericidal/permeability-increasing protein family, which includes the BPIFB3 gene, is essential for innate immunity [66,67]. Similarly, the TRIM (tripartite motif) family, including TRIM39.1, TRIM27.2, and TRIM27.1, plays significant roles in immune regulation, particularly in antiviral defense and apoptosis. These TRIM genes are associated with the Major Histocompatibility Complex (MHC), highlighting their importance in immune responses [68]. Both BPIFB3 and TRIM genes are essential for the activation of pro-inflammatory cytokines, and their decreased expression may facilitate the replication and persistence of Newcastle Disease Virus (NDV) within infected tissues [66,67,68].

In the current experiment, a decrease in the expression levels of BPIFB3 and TRIM genes was observed in the lungs of NDV-infected chicken embryos at 24 h post-infection (hpi). This downregulation may represent a strategy employed by the virus to evade the host’s immune response, thereby enhancing viral replication and spread within the host. Conversely, the increase in TRIM14 expression (1.5-fold change) suggested an active response to NDV infection [68], indicating that TRIM14 may play a role in initiating the modulation of the host’s defense mechanisms following infection at 24 hpi. Additionally, another study reported that the innate immune response remained both consistent and robust 72 h after NDV challenge in 18-day-old chicken embryos, emphasizing the dynamic nature of immune responses during viral infections [26]. This interplay between upregulated and downregulated genes underscores the complexity of host–pathogen interactions during NDV infection and highlights potential targets for enhancing disease resistance in poultry [67,68].

KLHL14 is highly expressed in infected embryos, and it was found to be in immune tissues, especially in B cells and involved in regulation of B cell regulation and stability [69]. The CYTB, CAV1, GATA3, HBBA, ND4, ND4L, and ND5 genes are crucial for various cellular functions, particularly in energy metabolism, immune responses, and cellular signaling [70]. During viral infections, viruses hijack and manipulate the host cell machinery to enhance their replication, which may lead to the suppression of genes associated with mitochondrial function and cellular metabolism. This includes mitochondrial genes that encode components of the electron transport chain [25,55]. The transcription factor GATA-3 is selectively expressed in Th2 cells and plays a critical role in Th2 differentiation and cytokines’ interleukin (IL) expressions [71,72]. Similarly, it was found that, in contrast to birds resistant to Marek’s disease (MD), the spleen of birds vulnerable to MD had much lower mitochondrial DNA levels throughout the transformation phase [73].

A recent study demonstrated that the chicken thymus exhibited significant and distinct expression of various innate immune genes, including NR1H4, RBM14, SLC26A6, SLC11A1, MASP2, CYBA, CATHB1, PTX3, MASP1, COLEC11, SOCS1L, OTOP1, COCH, TMEM173, CFD, APOA4, MIF, RARRES2, TRIM62, TKFC, SERPING1, HEXIM1, STAT2, FAU, NOP53, GFI1, and NLRX1. The responses of these genes were found to be conserved across different organisms [22]. The laryngotracheitis virus infection also caused differential expression of 789 genes in the lung cells of chicken embryos. These genes included those that regulate the cell cycle (cyclin B2, CDK1, and CKI3), the immune system (cytokines, chemokines, MHC, and NF), matrix metalloproteinases (MMPs), and cellular metabolism [40]. The HSP70 family of heat shock proteins is the most conserved across a wide range of species [48,74,75]. The increased expression of heat shock protein (HSP) genes, including HSPB8, HSPB9, HSPA2, and HSP90AA1, in the lungs of infected embryos, as compared to control, uninfected chicken embryos, was one of the findings of the current study. Interestingly, HSPB8 (P60), HSPA2 (HSP70), and HSP90AA1 genes were highly expressed in infected embryos of Kadaknath, followed by Aseel, compared to White Leghorn, which indicated the heat tolerance of the indigenous breed [76,77]. HSPs can exert antiviral effects by inhibiting viral replication through interactions with viral components and activating immune pathways to protect host cells. While their primary function is to maintain cellular homeostasis, some HSPs are hijacked by viruses to facilitate invasion, replication, and maturation, thereby enhancing viral survival under adverse conditions [78]. Targeting specific HSPs may enhance antiviral responses or reduce viral replication by disrupting the virus’ ability to exploit these essential cellular proteins. For example, HSP60 has been identified as a novel antiviral protein that inhibits viral replication, while the RNA-induced silencing complex (RISC) binds to viral mRNA to suppress translation [76]. In studies involving HPV, secreted HSP70 has been shown to effectively target dendritic cells with relevant antigens, enhancing antigen-specific immune responses [79]. Additionally, HSPs regulate immune signaling pathways to combat viral infections [78]. Furthermore, it was discovered that HSP60 may have interactions with mitochondrial antiviral signaling protein, a crucial signal transduction protein that triggers the synthesis of type I interferon [80].

### 4.2. GO and KEGG Pathway Analysis

Gene Ontology (GO) terms are increasingly valuable for predicting protein functions, though the number of terms used can be quite large. When predicting non-classical secretory proteins in eukaryotes and prokaryotes, the most informative GO terms are those associated with subcellular localization. A ranking of GO terms in this context revealed that the top terms in both datasets during the embryonic stage were associated with subcellular locations: GO:0005576 (extracellular region), GO:0005634 (nucleus), GO:0005737 (cytoplasm), GO:0005615 (extracellular space), GO:0016020 (membrane), GO:0005886 (plasma membrane), and GO:0008152 (metabolic process) [51,81]. Similarly, another study highlighted a strong presence of the KEGG pathways gga01100 (metabolic pathways) and gga04110 (cell cycle) during the early embryonic stage [51]. This suggests that embryos grow more quickly and have a high amount of metabolic activity [82], whereas, the downregulation of the BPGM (bisphosphoglycerate mutase) gene in the glycolysis/gluconeogenesis pathway (gga00010) during infection in chickens can lead to various physiological consequences that may alter metabolic activity in the embryo [83]. Further, the PPAR signaling pathway (gga03320) was found to have the highest expression of APOC3 and APOA1 genes, which are actively involved in lipid metabolism [84]. The apolipoprotein (C3 and A1) gene is responsible for producing lipoproteins, particularly triglyceride (TG)-rich lipoproteins and high-density lipoprotein (HDL) particles, respectively [85,86]. ApoA1 exhibits may anti-inflammatory and anti-apoptotic properties during Newcastle Disease Virus (NDV) infection [86]. Additionally, it has been found that lipoproteins produced in chicken lung tissue can inhibit the hemagglutination activity of the active Newcastle Disease Virus [87]. The gga03320 pathway gene promotes adipocyte differentiation to enhance blood glucose uptake [88]. Similarly, in the metabolic pathway, the highly expressed PLA2G12B gene, part of the phospholipase A2 (PLA2) family, encodes an enzyme that hydrolyzes phospholipids, influencing membrane fluidity and permeability, which can impact viral entry. Beyond facilitating viral invasion, PLA2 enzymes also modulate immune responses by altering host cell membranes, shaping the inflammatory environment in ways that can either support pathogen clearance or enhance infection [89].

Further, pathway analysis also highlighted the critical roles of cytokine production and Toll-like receptors (TLRs) in the immune response, particularly in recognizing pathogen-associated molecular patterns (PAMPs). Chicken TLRs have been shown to recognize a broad array of PAMPs, leading to enhanced immune responses. TLR21 in chickens can recognize immunostimulatory CpG-oligodeoxynucleotides, further emphasizing the role of TLRs in pathogen recognition [90,91]. The lung of a newly born chick was shown to have TLR21 transcript expression. TLR21 recognition is essential for activating innate immune cells, such as dendritic cells and natural killer (NK) cells, which release chemokines and cytokines to fight infections [90,91,92].

Cytokines are another important component of the immune system, acting as soluble extracellular proteins and glycoproteins that regulate intercellular interactions. They have a substantial pleiotropic effect on mobilizing cells engaged in both innate and adaptive immune responses, influencing processes such as inflammation, cell proliferation, cell differentiation, apoptosis, angiogenesis, and tissue repair to restore homeostasis. Cytokines allow communication and coordination between immune cells by acting on particular receptors on target cells, organizing a comprehensive response to varied stresses and maintaining general health [93,94]. Human extracellular vesicles have higher quantities of 11 cytokines, including IFNγ, IL2, IL4, IL12p70, IL17, IL21, IL22, IL33, ITAC, TGFβ, and TNFα, compared to their free form in most systems [95].

Previous reports indicated that TK1 plays a significant role in anti-apoptosis, DNA synthesis, and cell proliferation. When TK1 gene expression is downregulated, it results in increased apoptosis and a notable reduction in the proliferation of host cells [96]. This type of activation can lead to autophagy during Newcastle Disease Virus (NDV) infection, which may further downregulate genes involved in normal cellular functions, including carbonic anhydrases (CA2 and CA13), essential for maintaining cellular pH. Autophagy acts as a double-edged sword; while it aids in clearing viral particles, it can also suppress the expression of certain host genes [97].

In the context of chicken embryo development, pathway analysis identified several key processes essential for the embryo’s rapid growth and maturation. These processes include chromosome organization, mitosis, DNA replication, mitochondrial activity, and the roles of the CMG (Cdc45-MCM-GINS) and MCM (mini-chromosome maintenance) complexes. These processes are likely crucial in cell cycle and multiplication, occurring rapidly in the developing chicken embryo [98,99]. Numerous publications demonstrated the critical role that mitochondria and mtDNA play in the healthy embryonic development, with an abundant copy number of mtDNA [100,101]. Similarly, pathways of the current study also emphasized that there was increased mitochondrial activity during the embryonic stage, resulting in elevated ATP levels [82]. In chickens infected with MD virus, the ATP (6 and 8) genes, which encode subunits of Complex V, showed increased expression levels. On the other hand, in accordance with our findings, it was found that the ND (1, 2, 3, 4, 4L, 5, and 6) genes, which encode subunits of NADH dehydrogenase (Complex I), exhibited decreased expression levels in lymphoid tissue [73]. The processing, stability, and upkeep of the mitochondrial genome depend on the genes DNA2, MGME1, and SLC25A4. Reduced numbers of mitochondrial copies are often the result of changes in these genes’ transcripts [73,102,103,104,105]. Additionally, research has shown that NDV infection causes mitochondrial damage, which may lead to the downregulation of mtDNA genes [97].

Although the immune system of a chick embryo is still immature, it possesses active defense mechanisms mediated by cytokines and natural antimicrobial peptides that effectively combat invading microbes. In this study, we found that four host defense peptides (HDPs), including avian β-defensin (AvBD10 and AvBD13) and cathelicidin (CATH1 and CATH3), were highly expressed in infected chicken embryo lungs [45,106]. It has been observed that AvBDs and CATHs are expressed in different tissues, such as the bone marrow, multiple lymphoid organs, the respiratory system, and the gastrointestinal tract [106,107]. Their increased expression suggests a defensive mechanism activated in response to NDV infection.

By analyzing 150 differentially expressed genes across various pathways, we constructed a protein–protein interaction network and identified several key genes: APOC3 from the PPAR signaling pathway [84], HGD, HMGCS2, HPD, and FAH from the steroid biosynthesis pathway, and ALDOB from the glycolysis pathway, with higher topology scores ranging from 0.701 to 0.999 These scores indicate their critical roles in metabolic pathways during viral infections. Further, PPIs confirmed that the cyclin protein family, including CCNA2, CCNB1, CCNB3, and CCNE2, plays a crucial role in the regulation of the cell cycle by binding and activating cyclin-dependent kinases (CDKs), particularly CDK1 [108]. A recent report confirmed that the Gene Ontology functional enrichment analysis for the CDK1, CCNA2, and CCNB1 gene clusters and their neighboring genes primarily highlighted processes such as histone phosphorylation, chromosome segregation, regulation of ubiquitin protein ligase activity, organelle organization, cell division, and overall regulation of the cell cycle and nuclear division [109]. The under-expression of these genes results in the downregulation of the cell cycle pathway, which in turn may lead to reduced growth of the embryo. Similarly, MCM2–6 proteins are members of the mini-chromosome maintenance (MCM) complex, which has a critical role in DNA synthesis during cell division. These proteins are essential for both the initiation and elongation of DNA replication, functioning as a helicase that unwinds DNA strands. However, during Newcastle Disease Virus (NDV) infection, the expression of these genes is downregulated. This downregulation can impair DNA replication processes, potentially leading to reduced cell proliferation and growth in the affected tissues [110,111].

The immune response to viral infections is a quantitative trait influenced by multiple genes, necessitating the evaluation of significant DEGs on an individual basis. Understanding the specific effects of these genes on immune responses and altered metabolism during infection is crucial for identifying candidate genes that can be targeted for selection and breeding. This approach aims to enhance the resistance of poultry populations and improve production rates, thereby effectively addressing global food security challenges.

Moreover, recent studies suggested that in-ovo microbial challenges provide valuable insight into the cellular mechanisms affected by stress in newborn chicks. Findings from this research will support future investigations into metabolic, cellular, and immune signaling pathways, ultimately contributing to the development of more effective management strategies to enhance the health of chicken breeds.

## 5. Conclusions

This study investigated the alteration in transcript levels due to NDV infection in 18-day-old chicken embryos. The findings revealed significant alterations in gene expression, with 594 genes differentially expressed in infected embryos, including key immune and metabolic genes. Understanding these DEGs is essential for developing strategies to enhance viral resistance in chicken breeds. Key genes, such as APO, PLA2, BPIFB3, and members of the TRIM family, play significant roles during viral infections. Further investigation into these genes could lead to the identification of therapeutic or vaccine strategies aimed at strengthening the innate immune response by targeting these genes or their associated pathways. Additionally, the identified lncRNAs in NDV-infected embryos may be instrumental in regulating the host’s immune response, potentially serving as biomarkers for viral infections or targets for future therapeutic interventions. Exploring their specific functions could provide valuable insights into the molecular mechanisms underlying antiviral immunity in poultry. Furthermore, the research concurrently revealed that NDV infection significantly altered cellular, metabolic, mitochondrial, and mitotic activities. While this research primarily focused on the molecular impact of NDV in embryos, it is crucial to conduct additional trials in live adult birds to gain a comprehensive understanding of the NDV. Although studying immune responses in 18- to 19-day-old embryos provides valuable insights into early developmental immunity, adult birds exhibit fully developed immune systems that operate through distinct molecular mechanisms and respond differently to environmental stressors. Such studies are essential for effective management of the poultry sector and for addressing global food security challenges.

## Data Availability

The dataset created and analyzed in the current study will be made available upon reasonable request.

## Supplementary Materials

The following supporting information can be downloaded at: https://www.mdpi.com/article/10.3390/metabo14120669/s1, Figure S1: Principal Component Analysis (PCA) plot of normalized gene expression.; PCA is used to perform data reduction when there are a high number of variables (in this case genes). The algorithm will generate a few principle components which can account for the variation present in the variables. When the values of two major components (PC1, and PC2) are plotted, the samples that have similar variance will fall in the same plane of the graph. The X-axis represents PC1 and the Y-axis represents PC2. While reading the graph the % of variance the axis represents should be considered; Supplementary File S1: Table S1. List of primers used for validation of RNA sequencing result; Table S2. Summary of Raw sequence data and quality; Table S3. Read alignment statistics; Table S4. Number of expressed genes in each sample (genes with at least 1 mapped read); Table S5. Number of transcripts expressed from each chromosome; Supplementary File S2: Significant Differently Expressed Genes.
