# Supplementary material for: Transcriptomic Profiling Reveals Altered Expression of Genes Involved in Metabolic and Immune Processes in NDV-Infected Chicken Embryos
Source: Metabolites. 2024 Dec 2;14(12):669. doi: 10.3390/metabo14120669 (PMC11678133; doi:10.3390/metabo14120669)
Supplement: Supplementary file 1 [file metabolites-14-00669-s001.zip › Figure S1.pdf]

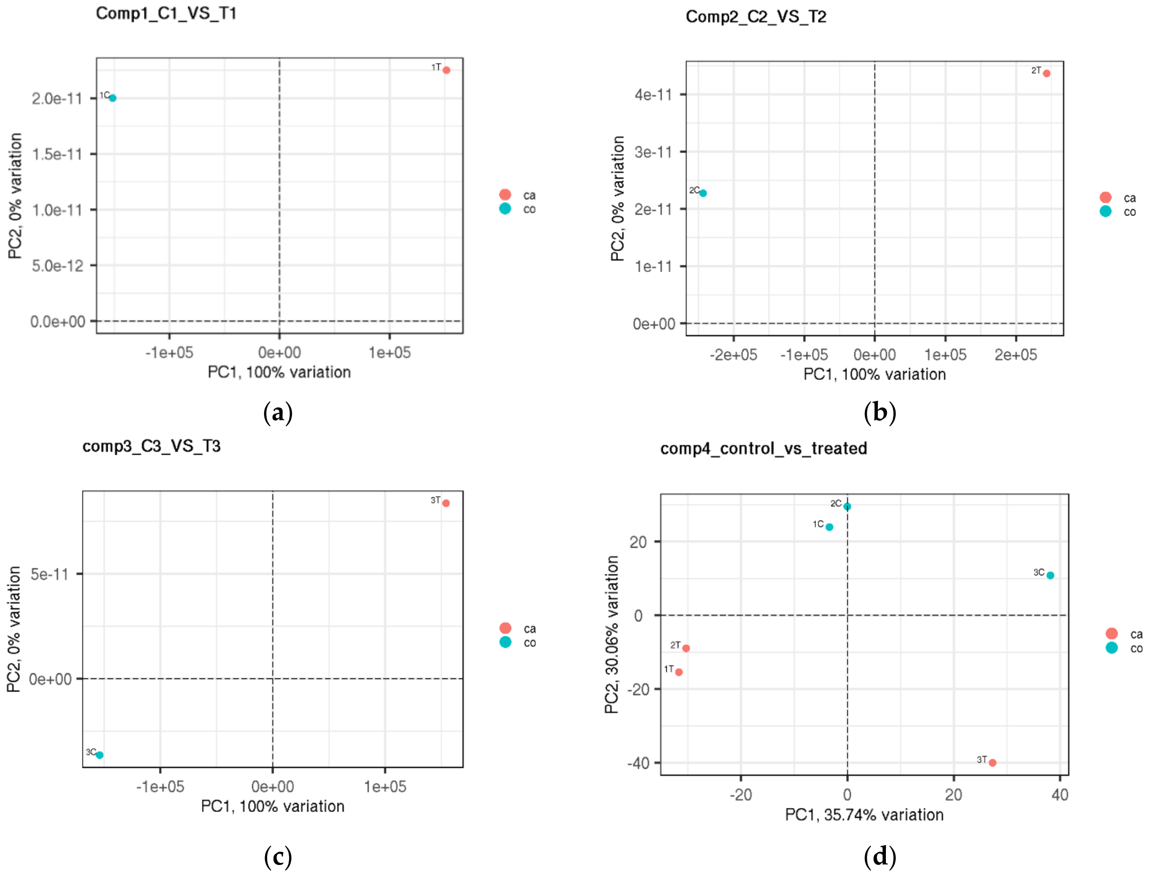

Figure S1. Principal Component Analysis (PCA) plot of normalized gene expression. PCA is used to perform data reduction when there are a high number of variables (in this case genes). The algorithm will generate a few principle components which can account for the variation present in the variables. When the values of two major components (PC1, and PC2) are plotted, the samples that have similar variance will fall in the same plane of the graph. The X-axis represents PC1 and the Y-axis represents PC2. While reading the graph the % of variance the axis represents should be considered.
