# Supplementary material for: Transcriptomic Profiling Reveals Altered Expression of Genes Involved in Metabolic and Immune Processes in NDV-Infected Chicken Embryos
Source: Metabolites. 2024 Dec 2;14(12):669. doi: 10.3390/metabo14120669 (PMC11678133; doi:10.3390/metabo14120669)
Supplement: Supplementary file 1 [file metabolites-14-00669-s001.zip › Supplementary File S1.pdf]

Table S1. List of primers used for validation of RNA sequencing result.

Gene Name	Primer sequence		Product size	References
	Forward Primer	Reverse Primer		
TLR2	CAAGACCTACCTGGAGTGGC	GGATGGCTACAGTCTCCATTTT	116	NM_001161650.3
TRIM14	GATTGGTGCAGCCTACCCTT	TCTCCCCCTTGTGAAATGCC	128	NM_001031431.2
IL1R2	TTCCCTCGCTCTTCTTCCCATTT	ATGGTGTGATCTGGGCAGTTT	137	XM_416914.8
NOS1	ATGCACAAACAGCAGGGAGT	CCAGGACAGGGATTGGGTTG	196	XM_040685201.2
GATA3	CCTTTGGACCTCACCATCCC	AAGCATTCAGCAGGGGAGTC	72	XM_046908462.1
β-Actin	TATGTGCAAGGCCGGTTTC	TGTCTTTCTGGCCCATACCAA	110	[38]

Table S2. Summary of Raw sequence data and quality.

Sample ID	Total reads	q20 rate	q30 rate	GC content
Aseel control	11712582	0.956	0.906	0.518
Aseel infected	19473512	0.959	0.911	0.520
Kadaknath control	27264370	0.958	0.907	0.549
Kadaknath infected	22294792	0.962	0.917	0.526
White Leghorn control	45866934	0.953	0.892	0.588
White Leghorn infected	27691980	0.957	0.910	0.532

`

Table S3. Read alignment statistics.

Sample ID	QC Passed Reads	Unique Mapped %	Multi Mapped %	Unmapped %
Aseel control	9665406	88.71	1.77	9.53
Aseel infected	16400020	88.81	1.71	9.48
Kadaknath control	20680458	86.49	1.98	11.53
Kadaknath infected	19415268	89.71	1.69	8.6
White Leghorn control	31375608	88.89	1.43	9.68
White Leghorn infected	23667448	88.2	1.69	10.11

Table S4. Number of expressed genes in each sample (genes with at least 1 mapped read).

Sample ID	Total Number of Genes	Expressed Genes
Aseel control	30108	17410
Aseel infected	30108	18733
Kadaknath control	30108	18502
Kadaknath infected	30108	18998
White Leghorn control	30108	20332
White Leghorn infected	30108	19915

Table S5. Number of transcripts expressed from each chromosome.

Chromosome No	lncRNA	Protein coding	Total number of transcripts
**Ch 1**	8	72	80
**Ch 2**	7	52	59
**Ch 3**	2	42	44
**Ch 4**	3	49	52
**Ch 5**	2	34	36
**Ch 6**	3	30	33
**Ch 7**	1	19	20
**Ch 8**	1	18	19
**Ch 9**	3	15	18
**Ch 10**	0	15	15
**Ch 11**	3	12	15
**Ch 12**	1	10	11
**Ch 13**	2	13	15
**Ch 14**	0	7	7
**Ch 15**	0	12	12
**Ch 16**	0	8	8
**Ch 17**	1	7	8
**Ch 18**	2	8	10
**Ch 19**	2	9	11
**Ch 20**	0	8	8
**Ch 21**	0	6	6
**Ch 22**	0	6	6
**Ch 23**	1	6	7
**Ch 24**	2	8	10
**Ch 25**	0	6	6
**Ch 26**	0	8	8
**Ch 27**	0	12	12
**Ch 28**	0	7	7
**Ch 30**	0	2	2
**Ch 31**	0	4	4
**Ch 34**	0	5	5
**Ch Z**	6	26	32
**Mitochondrial**	0	8	8
